# 4-Fluoro-2-[(*E*)-2-pyridyliminomethyl]phenol

**DOI:** 10.1107/S1600536809017280

**Published:** 2009-05-14

**Authors:** Ping Cui, Lei Shi

**Affiliations:** a Key Laboratory of Anhui Educational Department, Anhui University of Technology, Maanshan 243002, People’s Republic of China, and State Key Laboratory of Pharmaceutical Biotechnology, Nanjing University, Nanjing 210093, People’s Republic of China

## Abstract

In the title compound, C_12_H_9_FN_2_O, the dihedral angle between the benzene ring and the pyridine ring is 4.35 (16)°. The mol­ecular conformation is stabilized by an intra­molecular O—H⋯N hydrogen bond.

## Related literature

For a related structure, see: Li *et al.* (2006 ▶). For reference strutural data, see: Allen *et al.* (1987 ▶).
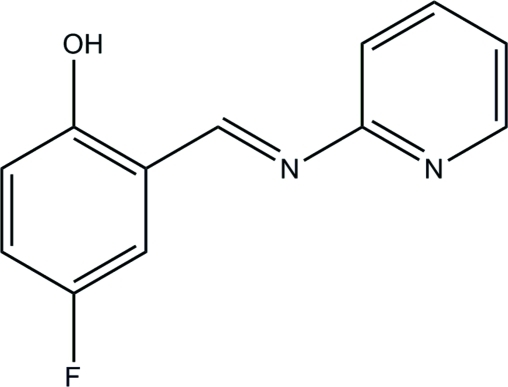


## Experimental

### 

#### Crystal data

C_12_H_9_FN_2_O
*M* *_r_* = 216.21
Monoclinic, 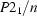

*a* = 13.1635 (11) Å
*b* = 6.2252 (6) Å
*c* = 13.8235 (17) Å
β = 113.33 (3)°
*V* = 1040.1 (3) Å^3^
*Z* = 4
Mo *K*α radiation
μ = 0.10 mm^−1^
*T* = 293 K
0.40 × 0.25 × 0.15 mm

#### Data collection

Enraf–Nonius CAD-4 diffractometer
Absorption correction: ψ scan (North *et al.*, 1968 ▶) *T*_min_ = 0.960, *T*_max_ = 0.985
1907 measured reflections
1825 independent reflections
1210 reflections with *I* > 2σ(*I*)
*R*_int_ = 0.064

#### Refinement

*R*[*F* ^2^ > 2σ(*F* ^2^)] = 0.053
*wR*(*F* ^2^) = 0.145
*S* = 1.05
1825 reflections
147 parameters
H-atom parameters constrained
Δρ_max_ = 0.16 e Å^−3^
Δρ_min_ = −0.17 e Å^−3^

### 

Data collection: *CAD-4 Software* (Enraf–Nonius, 1989 ▶); cell refinement: *CAD-4 Software*; data reduction: *XCAD4* (Harms & Wocadlo, 1995 ▶); program(s) used to solve structure: *SHELXS97* (Sheldrick, 2008 ▶); program(s) used to refine structure: *SHELXL97* (Sheldrick, 2008 ▶); molecular graphics: *SHELXTL* (Sheldrick, 2008 ▶); software used to prepare material for publication: *SHELXL97*.

## Supplementary Material

Crystal structure: contains datablocks global, I. DOI: 10.1107/S1600536809017280/hb2968sup1.cif

Structure factors: contains datablocks I. DOI: 10.1107/S1600536809017280/hb2968Isup2.hkl

Additional supplementary materials:  crystallographic information; 3D view; checkCIF report

## Figures and Tables

**Table 1:** Hydrogen-bond geometry (Å, °)

*D*—H⋯*A*	*D*—H	H⋯*A*	*D*⋯*A*	*D*—H⋯*A*
O1—H1⋯N1	0.82	1.86	2.588 (2)	147

**Fig. 1.**
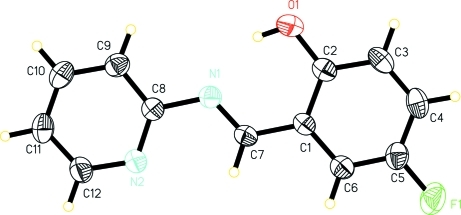
The molecular structure of (I) showing 30% probability displacement ellipsoids.

## supplementary crystallographic information

### Comment

Recently, we have reported the structural characterization of one Schiff base compound derived from the condensation of 5-chloro-salicylaldehyde and primary amines (Li *et al.*, 2006). As an extension of this work, we report here the crystal structure of the title compound, (I). In (I), all bond lengths are within normal ranges (Allen *et al.*, 1987) (Fig. 1). There is an intramolecular O—H···N hydrogen bond in (I). The dihedral angle between the two aromatic rings is 4.35(0.16)°.

### Experimental

Pyridin-2-amine (94 mg, 1 mmol) and 5-fluoro-salicylaldehyde (140 mg, 1 mmol) were dissolved in methanol (10 ml) at 323 K. The mixture was stirred for 2 h to give a clear yellow solution. After keeping the solution in air for 7 d by slow evaporation of the solvent, yellow blocks of (I) were formed at the bottom of the vessel, with 80% yield. The crystals were isolated, washed three times with methanol and dried in a vacuum desiccator containing anhydrous CaCl_2_.

### Refinement

All H atoms were positioned geometrically (C—H = 0.93–0.96 Å, O—H = 0.82Å) and refined as riding, with *U*_iso_(H) = 1.2*U*_eq_(carrier).

### Crystal data

**Table:**

C_12_H_9_FN_2_O	*F*(000) = 448
*M**_r_* = 216.21	*D*_x_ = 1.381 Mg m^−^^3^
Monoclinic, *P*2_1_/*n*	Mo *K*α radiation, λ = 0.71073 Å
Hall symbol: -P 2yn	Cell parameters from 25 reflections
*a* = 13.1635 (11) Å	θ = 9–12°
*b* = 6.2252 (6) Å	µ = 0.10 mm^−^^1^
*c* = 13.8235 (17) Å	*T* = 293 K
β = 113.33 (3)°	Block, yellow
*V* = 1040.1 (3) Å^3^	0.40 × 0.25 × 0.15 mm
*Z* = 4	

### Data collection

**Table:**

Enraf–Nonius CAD-4 diffractometer	1825 independent reflections
Radiation source: fine-focus sealed tube	1210 reflections with *I* > 2σ(*I*)
graphite	*R*_int_ = 0.064
ω/2θ scans	θ_max_ = 25.0°, θ_min_ = 1.8°
Absorption correction: ψ scan (North *et al.*, 1968)	*h* = 0→15
*T*_min_ = 0.960, *T*_max_ = 0.985	*k* = 0→7
1907 measured reflections	*l* = −16→15

### Refinement

**Table:**

Refinement on *F*^2^	Secondary atom site location: difference Fourier map
Least-squares matrix: full	Hydrogen site location: inferred from neighbouring sites
*R*[*F*^2^ > 2σ(*F*^2^)] = 0.053	H-atom parameters constrained
*wR*(*F*^2^) = 0.145	*w* = 1/[σ^2^(*F*_o_^2^) + (0.0657*P*)^2^ + 0.1652*P*] where *P* = (*F*_o_^2^ + 2*F*_c_^2^)/3
*S* = 1.05	(Δ/σ)_max_ < 0.001
1825 reflections	Δρ_max_ = 0.16 e Å^−^^3^
147 parameters	Δρ_min_ = −0.17 e Å^−^^3^
0 restraints	Extinction correction: *SHELXL97* (Sheldrick, 2008), Fc^*^=kFc[1+0.001xFc^2^λ^3^/sin(2θ)]^-1/4^
Primary atom site location: structure-invariant direct methods	Extinction coefficient: 0.089 (10)

### Special details

**Table:**

Geometry. All e.s.d.'s (except the e.s.d. in the dihedral angle between two l.s. planes) are estimated using the full covariance matrix. The cell e.s.d.'s are taken into account individually in the estimation of e.s.d.'s in distances, angles and torsion angles; correlations between e.s.d.'s in cell parameters are only used when they are defined by crystal symmetry. An approximate (isotropic) treatment of cell e.s.d.'s is used for estimating e.s.d.'s involving l.s. planes.
Refinement. Refinement of *F*^2^ against ALL reflections. The weighted *R*-factor *wR* and goodness of fit *S* are based on *F*^2^, conventional *R*-factors *R* are based on *F*, with *F* set to zero for negative *F*^2^. The threshold expression of *F*^2^ > σ(*F*^2^) is used only for calculating *R*-factors(gt) *etc*. and is not relevant to the choice of reflections for refinement. *R*-factors based on *F*^2^ are statistically about twice as large as those based on *F*, and *R*- factors based on ALL data will be even larger.

### Fractional atomic coordinates and isotropic or equivalent isotropic displacement parameters (Å^2^)

**Table:**

	*x*	*y*	*z*	*U*_iso_*/*U*_eq_	
C1	0.96836 (16)	0.2734 (3)	0.23488 (17)	0.0457 (6)	
C2	1.06679 (18)	0.2492 (4)	0.21918 (18)	0.0529 (6)	
C3	1.1349 (2)	0.0730 (4)	0.2622 (2)	0.0633 (7)	
H3	1.1996	0.0568	0.2510	0.076*	
C4	1.1080 (2)	−0.0767 (4)	0.3207 (2)	0.0658 (7)	
H4	1.1540	−0.1936	0.3496	0.079*	
C5	1.0116 (2)	−0.0506 (4)	0.3357 (2)	0.0619 (7)	
C6	0.94233 (19)	0.1189 (3)	0.29492 (19)	0.0548 (6)	
H6	0.8779	0.1318	0.3069	0.066*	
C7	0.89393 (17)	0.4506 (3)	0.18913 (17)	0.0484 (6)	
H7	0.8301	0.4632	0.2022	0.058*	
C8	0.83993 (18)	0.7650 (3)	0.08721 (17)	0.0488 (6)	
C9	0.8652 (2)	0.9050 (4)	0.02246 (19)	0.0591 (7)	
H9	0.9269	0.8816	0.0070	0.071*	
C10	0.7981 (2)	1.0788 (4)	−0.01873 (19)	0.0673 (7)	
H10	0.8141	1.1762	−0.0618	0.081*	
C11	0.7071 (2)	1.1067 (4)	0.0045 (2)	0.0677 (7)	
H11	0.6600	1.2231	−0.0223	0.081*	
C12	0.6870 (2)	0.9583 (4)	0.0684 (2)	0.0660 (7)	
H12	0.6247	0.9772	0.0835	0.079*	
F1	0.98419 (14)	−0.2003 (2)	0.39318 (14)	0.0938 (6)	
N1	0.91366 (15)	0.5906 (3)	0.13106 (15)	0.0514 (5)	
N2	0.75169 (16)	0.7888 (3)	0.11012 (15)	0.0582 (6)	
O1	1.09729 (14)	0.3937 (3)	0.16264 (16)	0.0721 (6)	
H1	1.0476	0.4818	0.1364	0.108*	

### Atomic displacement parameters (Å^2^)

**Table:**

	*U*^11^	*U*^22^	*U*^33^	*U*^12^	*U*^13^	*U*^23^
C1	0.0411 (11)	0.0435 (12)	0.0504 (13)	−0.0015 (10)	0.0159 (10)	−0.0095 (10)
C2	0.0488 (13)	0.0527 (13)	0.0569 (14)	−0.0022 (11)	0.0206 (11)	−0.0129 (12)
C3	0.0499 (14)	0.0678 (17)	0.0690 (16)	0.0094 (13)	0.0201 (13)	−0.0160 (14)
C4	0.0655 (17)	0.0503 (15)	0.0676 (17)	0.0120 (13)	0.0116 (13)	−0.0067 (13)
C5	0.0649 (16)	0.0453 (13)	0.0677 (16)	−0.0035 (12)	0.0181 (13)	0.0006 (12)
C6	0.0502 (13)	0.0500 (14)	0.0638 (15)	−0.0036 (11)	0.0221 (11)	−0.0062 (12)
C7	0.0439 (12)	0.0493 (13)	0.0539 (14)	−0.0027 (10)	0.0216 (11)	−0.0089 (11)
C8	0.0513 (13)	0.0479 (13)	0.0462 (13)	−0.0020 (11)	0.0181 (11)	−0.0058 (11)
C9	0.0651 (16)	0.0579 (15)	0.0577 (15)	−0.0051 (12)	0.0280 (13)	−0.0030 (12)
C10	0.0829 (19)	0.0613 (16)	0.0551 (15)	−0.0051 (15)	0.0248 (14)	0.0088 (13)
C11	0.0761 (18)	0.0572 (16)	0.0582 (15)	0.0115 (14)	0.0143 (14)	0.0083 (13)
C12	0.0631 (16)	0.0661 (16)	0.0678 (16)	0.0146 (13)	0.0249 (13)	0.0088 (14)
F1	0.1042 (13)	0.0645 (10)	0.1094 (13)	0.0031 (9)	0.0390 (10)	0.0279 (9)
N1	0.0525 (11)	0.0486 (11)	0.0553 (12)	−0.0021 (9)	0.0237 (9)	−0.0043 (10)
N2	0.0564 (12)	0.0585 (12)	0.0618 (13)	0.0107 (10)	0.0257 (10)	0.0087 (10)
O1	0.0616 (11)	0.0784 (13)	0.0899 (14)	0.0050 (9)	0.0444 (10)	0.0052 (11)

### Geometric parameters (Å, °)

**Table:**

C1—C6	1.399 (3)	C7—H7	0.9300
C1—C2	1.404 (3)	C8—N2	1.328 (3)
C1—C7	1.444 (3)	C8—C9	1.381 (3)
C2—O1	1.353 (3)	C8—N1	1.422 (3)
C2—C3	1.392 (3)	C9—C10	1.370 (3)
C3—C4	1.369 (3)	C9—H9	0.9300
C3—H3	0.9300	C10—C11	1.368 (4)
C4—C5	1.375 (3)	C10—H10	0.9300
C4—H4	0.9300	C11—C12	1.375 (3)
C5—C6	1.362 (3)	C11—H11	0.9300
C5—F1	1.363 (3)	C12—N2	1.335 (3)
C6—H6	0.9300	C12—H12	0.9300
C7—N1	1.279 (3)	O1—H1	0.8200
			
C6—C1—C2	118.4 (2)	C1—C7—H7	119.2
C6—C1—C7	120.12 (19)	N2—C8—C9	122.8 (2)
C2—C1—C7	121.5 (2)	N2—C8—N1	119.8 (2)
O1—C2—C3	118.8 (2)	C9—C8—N1	117.3 (2)
O1—C2—C1	121.4 (2)	C10—C9—C8	119.1 (2)
C3—C2—C1	119.7 (2)	C10—C9—H9	120.4
C4—C3—C2	121.0 (2)	C8—C9—H9	120.4
C4—C3—H3	119.5	C11—C10—C9	118.9 (2)
C2—C3—H3	119.5	C11—C10—H10	120.6
C3—C4—C5	118.6 (2)	C9—C10—H10	120.6
C3—C4—H4	120.7	C10—C11—C12	118.4 (2)
C5—C4—H4	120.7	C10—C11—H11	120.8
C6—C5—F1	118.9 (2)	C12—C11—H11	120.8
C6—C5—C4	122.4 (2)	N2—C12—C11	123.8 (2)
F1—C5—C4	118.8 (2)	N2—C12—H12	118.1
C5—C6—C1	119.8 (2)	C11—C12—H12	118.1
C5—C6—H6	120.1	C7—N1—C8	120.91 (19)
C1—C6—H6	120.1	C8—N2—C12	117.0 (2)
N1—C7—C1	121.6 (2)	C2—O1—H1	109.5
N1—C7—H7	119.2		
			
C6—C1—C2—O1	−179.5 (2)	C6—C1—C7—N1	−177.70 (19)
C7—C1—C2—O1	1.6 (3)	C2—C1—C7—N1	1.2 (3)
C6—C1—C2—C3	0.7 (3)	N2—C8—C9—C10	1.3 (3)
C7—C1—C2—C3	−178.2 (2)	N1—C8—C9—C10	−178.0 (2)
O1—C2—C3—C4	179.5 (2)	C8—C9—C10—C11	−0.9 (4)
C1—C2—C3—C4	−0.6 (3)	C9—C10—C11—C12	0.0 (4)
C2—C3—C4—C5	0.4 (4)	C10—C11—C12—N2	0.6 (4)
C3—C4—C5—C6	−0.2 (4)	C1—C7—N1—C8	−179.53 (19)
C3—C4—C5—F1	179.5 (2)	N2—C8—N1—C7	2.8 (3)
F1—C5—C6—C1	−179.4 (2)	C9—C8—N1—C7	−177.8 (2)
C4—C5—C6—C1	0.3 (4)	C9—C8—N2—C12	−0.8 (3)
C2—C1—C6—C5	−0.5 (3)	N1—C8—N2—C12	178.5 (2)
C7—C1—C6—C5	178.4 (2)	C11—C12—N2—C8	−0.2 (4)

### Hydrogen-bond geometry (Å, °)

**Table:**

*D*—H···*A*	*D*—H	H···*A*	*D*···*A*	*D*—H···*A*
O1—H1···N1	0.82	1.86	2.588 (2)	147
